# Structural Studies of the Lipopolysaccharide from the Fish Pathogen *Aeromonas veronii* Strain Bs19, Serotype O16

**DOI:** 10.3390/md12031298

**Published:** 2014-03-07

**Authors:** Anna Turska-Szewczuk, Katarzyna A. Duda, Dominik Schwudke, Agnieszka Pekala, Alicja Kozinska, Otto Holst

**Affiliations:** 1 Department of Genetics and Microbiology, M. Curie-Sklodowska University, Akademicka 19, Lublin 20-033, Poland; 2 Division of Structural Biochemistry, Research Center Borstel, Leibniz-Center for Medicine and Biosciences, Parkallee 4a/c, Borstel D-23845, Germany; E-Mails: kduda@fz-borstel.de (K.A.D); oholst@fz-borstel.de (O.H.); 3 Division of Bioanalytical Chemistry, Research Center Borstel, Leibniz-Center for Medicine and Biosciences, Parkallee 10, Borstel D-23845, Germany; E-Mail: dschwudke@fz-borstel.de; 4 Department of Fish Diseases, National Veterinary Research Institute, Partyzantow 57, Pulawy 24-100, Poland; E-Mails: a.pekala@piwet.pulawy.pl (A.P.); koala@piwet.pulawy.pl (A.K.)

**Keywords:** lipopolysaccharide, *O*-specific polysaccharide, *Aeromonas veronii*, fish pathogen, ESI MS, NMR

## Abstract

Chemical analyses, mass spectrometry, and NMR spectroscopy were applied to study the structure of the lipopolysaccharide (LPS) isolated from *Aeromonas veronii* strain Bs19, serotype O16. ESI-MS revealed that the most abundant LPS glycoforms have tetra-acylated or hexa-acylated lipid A species, consisting of a bisphosphorylated GlcN disaccharide with an AraN residue as a non-stoichiometric substituent, and a core oligosaccharide composed of Hep_5_Hex_3_HexN_1_Kdo_1_P_1_. Sugar and methylation analysis together with 1D and 2D ^1^H and ^13^C NMR spectroscopy were the main methods used, and revealed that the *O*-specific polysaccharide (OPS) of *A. veronii* Bs19 was built up of tetrasaccharide repeating units with the structure: →4)-α-d-Qui*p*3NAc-(1→3)-α-l-Rha*p*-(1→4)-β-d-Gal*p*-(1→3)-α-d-Gal*p*NAc-(1→. This composition was confirmed by mass spectrometry. The charge-deconvoluted ESI FT-ICR MS recorded for the LPS preparations identified mass peaks of *SR*- and *R*-form LPS species, that differed by Δm = 698.27 u, a value corresponding to the calculated molecular mass of one OPS repeating unit (6dHexNAc6dHexHexHexNAc-H_2_O). Moreover, unspecific fragmentation spectra confirmed the sequence of the sugar residues in the OPS and allowed to assume that the elucidated structure also represented the biological repeating unit.

## 1. Introduction

*Aeromonas* spp. bacteria are widespread in aquatic environments and soil habitats and are also frequently isolated from raw and processed food. They are either mesophilic, motile, or psychrophilic non-motile Gram-negative rods [1,2,3]. *Aeromonas* strains identified as members of the gut microflora in fish and other aquatic animals (amphibians, reptiles) may cause various diseases under environmental stress conditions (overcrowding, poor water quality, organic pollution, and hypoxia) [4,5]. Amongst mesophilic and motile species, *A. hydrophila*, *A. caviae*, *A. sobria*, and *A. veronii* have been described as important fish pathogens. They cause chronic disease with open dermal ulcers and other pathological lesions or acute systemic infection referred to as motile aeromonad septicemia (MAS) [5,6,7].

These bacteria, especially belonging to the species *A. hydrophila*, *A*. *caviae*, and *A*. *veronii* bv. *sobria*, often have been associated with several categories of human infections. Clinical presentations of such diseases comprise both gastrointestinal, frequently foodborne diseases, and life-threatening extraintestinal infections, including septicemia, wound and urinary tract infections, and, occasionally, meningitis, especially in immunocompromised patients and children [5,8,9,10,11], however, recent data indicates that *Aeromonas* may also be primary causes of infections in immunocompetent hosts [12].

The possible mechanisms of *Aeromonas* infections are complex and multifactorial. Several extracellular products of *Aeromonas* including hemolysins, cytotonic and cytotoxic enterotoxins, proteases, lipases, and leucocidins have been suggested as possible contributory factors in the pathogenesis of these bacteria [12]. Amongst these, a type II secretion system (secretion of enterotoxin-Act) and a type III secretion system (T3SS) seem to be leading [10]. Recently characterized effectors of the type VI secretion system had actin-ADP ribosylation activity that induced host cell cytotoxicity [5,10].

Moreover, cell-surface components such as outer membrane proteins, lipopolysaccharide (LPS), S-layer, polar flagella, and pili (type IV and bundle-forming pili) have been identified as *Aeromonas* putative virulence factors [13,14,15,16]. An equally important non-fimbrial adhesion factors that have been implicated in the pathogenesis of *Aeromonas* spp. are S-layer and LPS. The S-layer enhances certain physical attributes of the bacterium, including increases in cellular hydrophobicity, cell aggregation, and cell-to-tissue adhesion [17]. As an adhesine, *S*-form LPS is indispensable for initial attachment of bacteria to host tissue and necessary during infection events, where it protects bacteria from complement-mediated killing and antimicrobial peptides [5]. It is plausible that some virulence factors located in the outer membrane require the presence of O-antigens (*O*-specific polysaccharides, OPS) for proper expression or functionality. In addition, the OPS variations seem to play an essential role at several stages of the infection process, including the adherence step and the ability to protect against host defense mechanisms [5]. Although it was not clearly evidenced which structural determinants are the most important for virulence, it was found that some O serotypes are more frequently associated with certain infections. Studies demonstrated that *Aeromonas* strains belonging to serogroups O11, O16, O18, and O34 (Sakazaki and Shimada scheme [18]) are associated with most cases of bacteremia, implying the OPS variants are relevant in systemic disease pathogenesis [5]. It was proven that smooth LPS was implicated to promote adhesion of *A. veronii* bv. *sobria* to HEp-2 cells, and switching off of the OPS by using specific monoclonal antibodies impaired bacterial adherence [19,20].

The species *A*. *veronii*, originally described by Hickman-Brenner *et al.* (1987), as a novel member of the genus is commonly diarrhea-associated and is a rare cause of bacteremia [21]. However, there were also reports of *A. veronii* bv. *veronii* and *A. veronii* bv. *sobria* septicemia incidences in immunocompromised patients [22,23]. Nevertheless, this species is commonly known as fish pathogen, especially associated with ulcerative syndrome [6,7]. In the light of the increased *Aeromonas* infection incidence rate and the economic importance of these diseases in fish farms as well as possible public health effects, it is essential to characterize the virulence factors of these bacteria.

In the majority of the *Aeromonas* strains studied, the OPS has a heteropolymeric repeating unit and contains aminohexoses and amino-6-deoxyhexoses as well as acidic non-carbohydrate substituents such as 3-hydroxy butyrate [24,25,26,27]. Recently, two new structures of OPS were established for the species *A. bestiarum*, which strains are frequently isolated in the course of motile aeromonad septicemia in Polish commercial ponds [28,29,30].

Now we report on the chemical structure of the LPS from *A. veronii* strain Bs19 serologically classified as serogroup O16, which was isolated from skin of carp (*Cyprinus carpio* L.) with hemorrhagic and necrotic ulcers [31].

## 2. Results and Discussion

### 2.1. Isolation of LPS and SDS-PAGE

**Figure 1:**
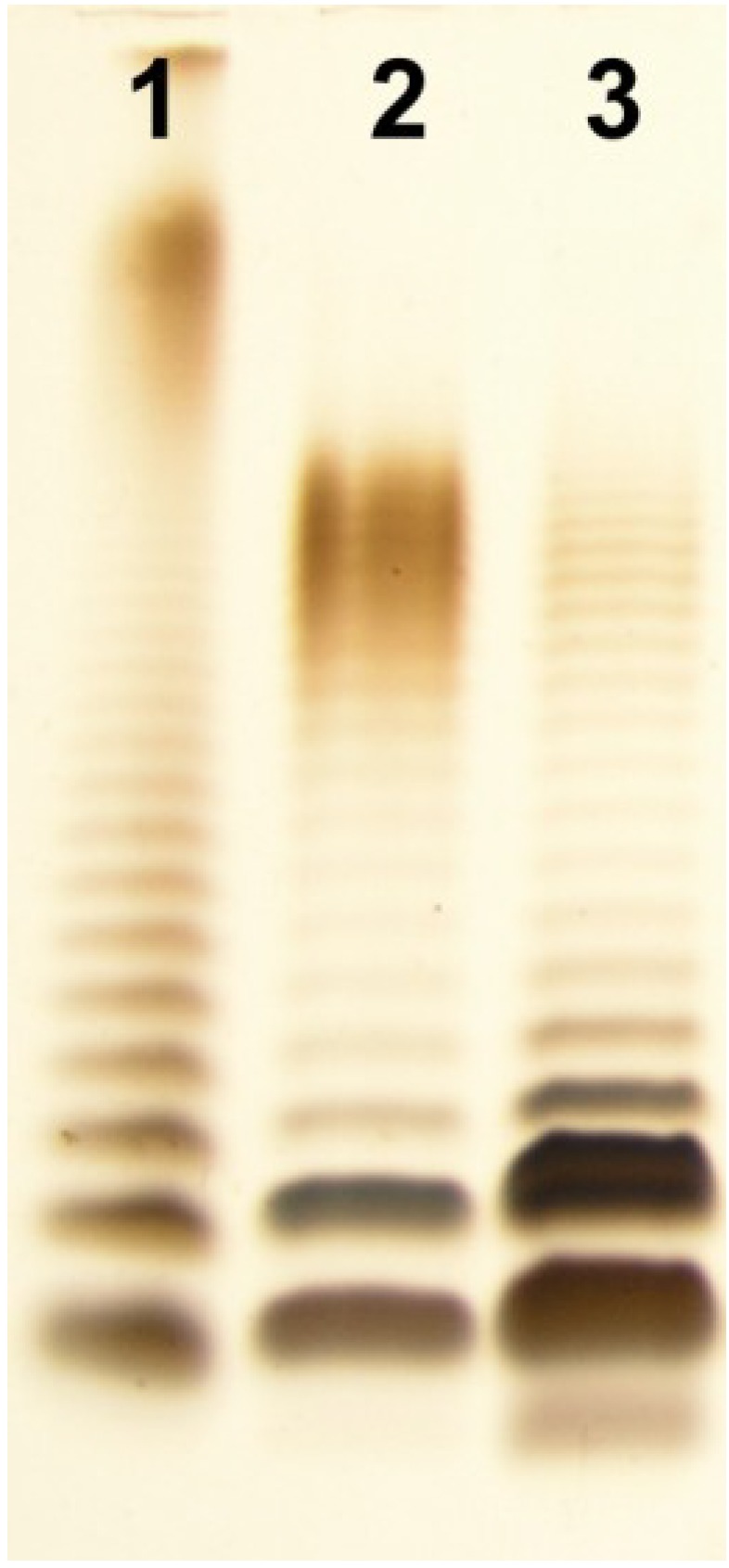
Silver-stained SDS-Tricine PAGE of the water- and phenol-soluble LPS fractions of *A. veronii* strain Bs19 (lane 2, lane 3, respectively), and *Salmonella enterica* sv. Typhimurium as reference (lane 1). Two micrograms were loaded per lane.

*A. veronii* Bs19 LPS was isolated by hot phenol-water extraction [32] from enzymatically digested bacterial cells [29]. It was found that the LPS species distributed between the water and phenol phases as hydrophilic and hydrophobic fractions, in yields of 4.2% and 0.3% of the dry bacterial cell mass, respectively. The SDS-PAGE analysis (Figure 1) of these preparations revealed that the smooth, *S*-form LPS species depicting a typical ladder-like pattern were recovered mainly from the water phase, and the rough *R*- or semi-rough *SR*-LPS species from the phenol phase. The yield of the hydrophobic LPS fraction, extracted from the phenol, which was less than 10% of the yield obtained from water, indicated a lower content of *R*-form LPS molecules in the cell envelope of *A. veronii* Bs19.

### 2.2. Chemical and ESI FT-ICR Mass Spectrometric Analyses of LPS

Sugar analyses of the LPS preparations were performed by GC-MS of the alditol acetates. The *R*- and *SR*-form LPS species contained Glc, GlcN, d,d-Hep, and l,d-Hep in a molar ratio of approx. 2.9:0.9:2.6:1. All these sugars (in a molar ratio of approx. 3.3:1:4.0:1) were also found in the fraction obtained from the water phase, which contained high molecular mass *S*-form LPS species. Additionally, the chemical analysis of both preparations showed 6-deoxyhexose (Rha), 3-amino-3,6-dideoxyglucose (Qui3N), Gal and GalN, in a molar ratio of approx. 1.2:1:1.2:1.0. These sugars were identified as components of the OPS (see Section 2.3). Kdo (3-deoxy-d-*manno*-2-octulosonic acid)—the only acidic sugar—was found in both LPS fractions. GC-MS analysis of the fatty acids as methyl esters and *O*-TMS derivatives identified 3-hydroxy myristic [14:0(3-OH)] and dodecanoic (12:0) acids, as the most abundant species. GlcN was identified as the sugar component of the lipid A. 

**Figure 2:**
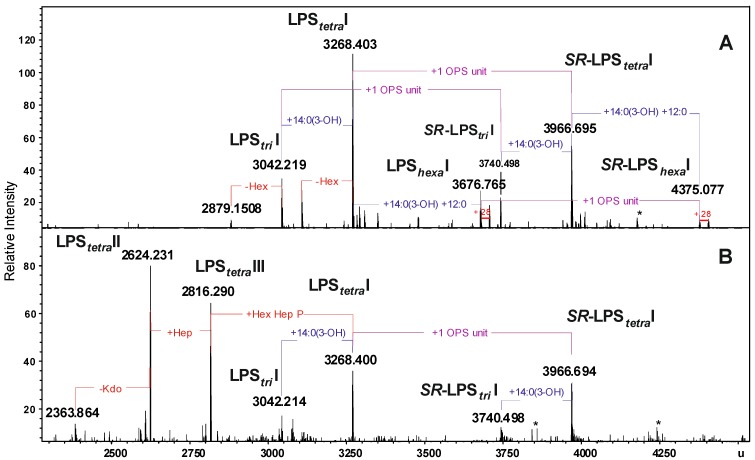
Charge-deconvoluted ESI FT-ICR mass spectra (negative ion mode) of the water- and phenol-soluble LPS fractions from *A. veronii* strain Bs19 (**A**) and (**B**), respectively. Indicated mass values refer to the monoisotopic signals of the neutral molecules. LPS*_tri_*, LPS*_tetra_*, LPS*_hexa_*, acylation state of the lipid A, * undefined contaminations.

The LPS preparations from *A. veronii* Bs19 were analyzed by ESI FT-ICR MS. The charge-deconvoluted ESI MS (negative-ion mode) (Figure 2A,B) of both LPS fractions showed a complex pattern of molecules originating from heterogeneity of lipid A and the core oligosaccharide. The heterogeneity was caused by non-stoichiometric substitutions with hexose (Hex, Δm = 162.05 u), heptose (Hep, Δm = 192.06 u), one or two fatty acid residues, 14:0(3-OH) (Δm = 226.19 u), and 14:0(3-OH) + 12:0 (Δm = 408.36 u), respectively, as well as peaks originating from different acyl chain length (*i.e.*, 12:0 and 14:0).

The mass spectra of LPS preparations showed a molecular peak at 3268.403 u corresponding to a *R*-LPS glycoform with the core decasaccharide -Hep_5_Hex_3_HexN_1_Kdo_1_P_1_ linked to tetra-acylated lipid A (LPS*_tetra_*I), which possessed a bisphosphorylated diglucosaminyl backbone (Figure 2, Table 1).

**Table 1:** Composition of the main species present in the charge deconvoluted ESI FT-ICR mass spectra (negative ion mode) of the water and phenol-soluble lipopolysaccharide (LPS) fractions of *A. veronii* Bs19.

Species	M_measured_ _water phase_	M_measured_ _phenol phase_	M_calculated_	Composition
**LPS*_tri_*I**	3042.219	3042.214	3042.203	Hep_5_Hex_3_HexN_3_KdoP_3_[14:0(3-OH)]_2_12:0
**LPS*_tetra_*I**	3268.403	3268.400	3268.397	Hep_5_Hex_3_HexN_3_KdoP_3_[14:0(3-OH)]_3_12:0
**LPS*_hexa_*I**	3676.765	3676.765	3676.757	Hep_5_Hex_3_HexN_3_KdoP_3_[14:0(3-OH)]_4_(12:0)_2_
***SR*-LPS*_tri_*I**	3740.498	3740.498	3740.480	6dHex6dHexNHep_5_Hex_4_HexN_4_KdoP_3_Ac_2_[14:0(3-OH)]_2_12:0
***SR*-LPS*_tetra_*I**	3966.695	3966.694	3966.671	6dHex6dHexNHep_5_Hex_4_HexN_4_KdoP_3_Ac_2_[14:0(3-OH)]_3_12:0
***SR*-LPS*_hexa_*I**	4375.077	4375.077	4375.031	6dHex6dHexNHep_5_Hex_4_HexN_4_KdoP_3_Ac_2_[14:0(3-OH)]_4_(12:0)_2_
**LPS*_tetra_*II**	−	2816.290	2816.300	Hep_4_Hex_2_HexN_3_KdoP_2_[14:0(3-OH)]_3_12:0-H_2_O
**LPS*_tetra_*III**	−	2624.231	2624.240	Hep_3_Hex_2_HexN_3_KdoP_2_[14:0(3-OH)]_3_12:0-H_2_O

Moreover, the spectra showed signals at 3740.498 and 3966.695 u (Figure 2), which corresponded to the semi-rough LPS glycoforms: *SR*-LPS*_tri_*I and *SR*-LPS*_tetra_*I, respectively, with different acylation patterns of the lipid A and carrying one OPS repeating unit (6dHexNAc6dHexHexHexNAc) minus H_2_O (calculated mass 698.274 u). The measured mass difference of Δm = 698.27 u was in full accordance with the chemical structure determined by NMR spectroscopy (see Section 2.3). Exclusively, the spectrum of the water-soluble fraction indicated signals at 3676.765 and 4375.077 u attributed to LPS glycoforms with hexa-acylated lipid A, namely *R*-LPS*_hexa_*I and *SR*-LPS*_hexa_*I, respectively. On the other hand, in the mass spectrum of the phenol-soluble LPS, two intensive signals at 2624.231 and 2816.290 u were assigned to the *R*-LPS glycoforms with tetra-acylated lipid A and a shorter core region being hepta- and octasaccharide, respectively (Figure 2, Table 1). 

**Figure 3:**
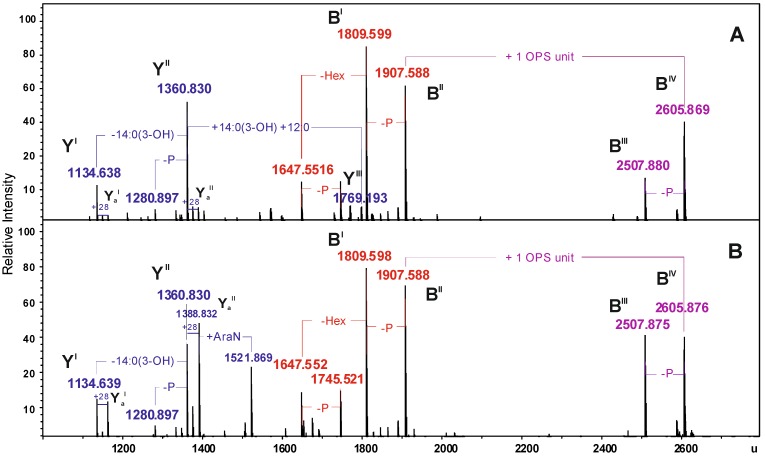
Charge-deconvoluted ESI FT-ICR mass spectra (negative ion mode) of the water- and phenol-soluble LPS fractions from *A. veronii* Bs19 with unspecific fragmentation in the collision cell (collision voltage 30 V) (**A**) and (**B**), respectively, providing the fragmentation of the labile linkage between the Kdo and lipid A. Mass numbers given refer to the monoisotopic masses of the neutral molecules.

For a more detailed interpretation, both LPS preparations were unspecifically fragmented in the collision cell which yielded Y- and B-mass fragments arising from cleavage of the labile ketosidic linkage between lipid A and Kdo of the core oligosaccharide (Figure 3) [33].

In particular, the mass spectra showed, e.g., signals at 1907.588 and 1809.599 u, which corresponded to the B-fragments originating from the core glycoforms and Y-fragments at 1360.830 and 1134.638 u derived from the lipid A species. In addition to these, a further B-fragment at 2605.869 u was observed, which was attributed the core oligosaccharide with one O-antigen repeating unit attached (Table 2).

Based on the chemical component analysis of main fragments, e.g., Y^II^ and B^II^ as well as Y^II^ and B^IV^, using high-resolution mass spectrometric data, the molecular composition of the LPS glycoforms, *R*-LPS*_tetra_*I and *SR*-LPS*_tetra_*I, respectively, was confirmed.

**Table 2:** Composition of the main Y- and B-fragments present in the charge deconvoluted ESI FT-ICR mass spectra (negative ion mode) of the water- and phenol-soluble LPS fractions from *A. veronii* strain Bs19 obtained with unspecific fragmentation.

Species	M_measured_ _water phase_	M_measured_ _phenol phase_	M_calculated_	Composition
**Y^I^**	1134.638	1134.639	1134.634	HexN_2_P_2_[14:0(3-OH)]_2_12:0
**Y_a_^I^**	1162.673	1162.672	1162.665	HexN_2_P_2_[14:0(3-OH)]_2_14:0
**Y^II^**	1360.830	1360.830	1360.827	HexN_2_P_2_[14:0(3-OH)]_3_12:0
**Y_a_^II^**	1388.831	1388.832	1388.859	HexN_2_P_2_[14:0(3-OH)]_3_14:0
**Y^III^**	1769.193	−	1769.188	HexN_2_P_2_[14:0(3-OH)]_4_(12:0)_2_
**B^I^**	1809.599	1809.598	1809.598	Hep_5_Hex_3_HexNKdo-2H_2_O
**B^II^**	1907.588	1907.588	1907.569	Hep_5_Hex_3_HexNKdoP-H_2_O
**B^III^**	2507.880	2507.875	2507.866	6dHex6dHexNHep_5_Hex_4_HexN_2_KdoAc_2_-2H_2_O
**B^IV^**	2605.869	2605.876	2605.843	6dHex6dHexNHep_5_Hex_4_HexN_2_KdoPAc_2_-H_2_O

### 2.3. Structural Studies of the OPS

The OPS was released from the water-soluble LPS fraction by mild-acid degradation followed by gel-permeation chromatography (GPC). Sugar analysis of the OPS (GC-MS of the alditol acetates) revealed Rha, Qui3N, Gal, and GalN in a relative peak area ratio of approx. 1:0.9:1.1:1.2. Qui3N was identified by comparing its retention time and mass spectra with those compounds obtained from the OPS of *E. coli* O5 [34] (a strain kindly provided from the Institute of Immunology and Experimental Therapy, Wroclaw, Poland) and *Aeromonas veronii* bv. *sobria* strain K49 [35]. The absolute configuration of monosaccharides determined by GLC of the acetylated (*S*)-2-butyl glycosides [36] identified the configuration of Rha as l and that of the other sugars as d.

Linkage analysis by GC-MS of the partially methylated alditol acetates derived from the methylated polysaccharide resulted in identification of 3-substituted Rha*p*, 4-substituted Qui*p*3N, 3-substituted Gal*p*N, and 4-substituted Gal*p*.

**Figure 4:**
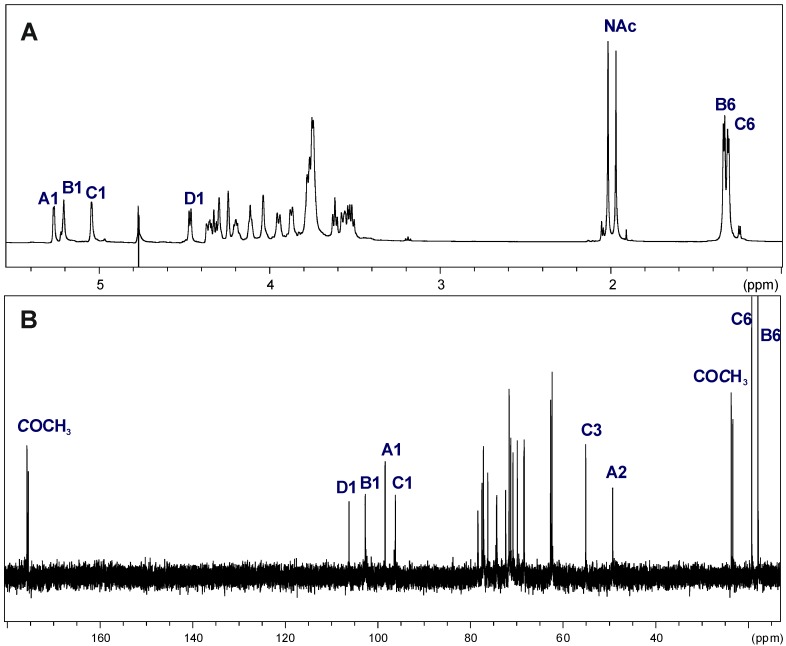
^1^H NMR (700.43 MHz) (**A**) and ^13^C NMR (176.14 MHz) (**B**) spectra of the OPS from *A. veronii* strain Bs19. Capital letters and Arabic numerals refer to atoms in the sugar residues denoted as shown in Table 3. NAc, N-acetyl groups, spectra were recorded at 32 °C in D_2_O as a solvent, relative to external acetone as reference (δ_H_ 2.225, δ_C_ 31.45).

The ^1^H and ^13^C NMR spectra showed that the OPS of *A. veronii* strain Bs19 had a regular structure composed of linear tetrasaccharide repeating units. The ^1^H NMR spectrum of the OPS (Figure 4A) contained signals for four anomeric protons at δ 5.27, 5.21, 5.05, and 4.47, labeled **A** through **D**, respectively. In the high field region of the spectrum, there were also signals originating from the methyl groups of 6-deoxysugars (Rha and Qui3N) at δ 1.33 and 1.30, and two signals of *N*-acetyl groups at δ 2.02 and 1.97.

The ^13^C NMR spectrum (Figure 4B) of the OPS contained signals for four anomeric carbons at δ 96.03 (**C**), 98.11 (**A**), 102.46 (**B**), and 106.02 (**D**); signals for two nitrogen-bearing carbons at δ 49.15 and 55.09 (GalN C-2 and Qui3N C-3, respectively); two methyl groups of 6-deoxysugars at δ 18.0 and 19.0 (Rha and Qui3N); *N*-acetyl groups (CH_3_ at δ 23.7 and CO at δ 175.6, and δ 23.2 and CO at δ 175.4, for GalN and Qui3N, respectively), and other non-anomeric sugar ring carbons in the region δ 68.24–78.33, some of which overlapped. The ^13^C NMR data showed that all the sugar residues were in the pyranose form [37] as no signals for ring carbons above δ 80, diagnostic of furanose, were detected.

The anomeric configuration of each monosaccharide was assigned on the basis of the ^3^*J*_H-1,H-2_ (measured on the DQF-COSY spectrum) and ^1^*J*_C-1,H-1_ coupling constants, and the *intra*-residual NOE contacts identified in the ROESY spectrum, whereas the ring configuration of each residue was inferred by the vicinal ^3^*J*_H,H_ coupling constants [37].

Chemical shifts of each spin system were assigned in ^1^H,^1^H, TOCSY, DQF-COSY, ROESY, ^1^H,^13^C HSQC, and ^1^H,^13^C HMBC experiments. All chemical shifts are summarized in Table 3.

Based on these data, the spin systems were assigned to four residues, one Rha*p*, one Qui*p*3NAc, one Gal*p*, and one Gal*p*NAc. In particular, the spin systems **A** (^3^*J*_1,2_ ~3.2 Hz) and **D** (^3^*J*_1,2_ ~8 Hz) were identified as α-Gal*p*NAc and β-Gal*p* residues, respectively [38]. A small *J*_1,2_ coupling constant ~3.5 Hz and chemical shifts of H-1 (δ 5.05) and C-5 (δ 68.24) indicated that the spin system **C** was α-linked Qui*p*3NAc [39]. The chemical shifts for H-5 and C-5 at δ_H_ 3.78 and δ_C_ 70.71, respectively, as well as signals of H-6 at δ_H_ 1.33 from the methyl group indicated that the spin system **B** was α-linked Rha*p* [40,41].

**Table 3:** ^1^H and ^13^C NMR chemical shifts of the constituents of the *O*-specific polysaccharides (OPS) of *A. veronii* strain Bs19. Spectra were recorded in D_2_O relative to external acetone as reference (δ_H_ 2.225, δ_C_ 31.45).

	Chemical Shifts (ppm)						
Sugar Residue		H-1	H-2	H-3	H-4	H-5	H-6
		C-1	C-2	C-3	C-4	C-5	C-6
→3)-α-d-Gal*p*NAc-(1→	**A**	5.27	4.35	3.95	4.24	4.11	3.75
		98.11	49.15	78.33	69.72	72.30	62.30
→3)-α-l-Rhal*p*-(1→	**B**	5.21	4.28	3.87	3.61	3.78	1.33
		102.46	68.34	77.14	71.21	70.71	18.00
→4)-α-d-Qui*p*3NAc-(1→	**C**	5.05	3.56	4.33	3.52	4.20	1.30
		96.03	71.50	55.09	77.44	68.24	19.00
→4)-β-d-Gal*p*-(1→	**D**	4.47	3.54	3.77	4.03	3.74	3.75
		106.02	71.45	74.17	77.04	76.06	62.30

Chemical shifts for NAc were δ_H_ 2.02 and δ_C_ 23.7/175.6 for **A** and δ_H_ 1.97 and δ_C_ 23.2/175.4 for **C**.

The *galacto* configuration of **A** and **D** was determined by the small ^3^*J*_3,4_ (~3 Hz) and ^3^*J*_4,5_ (~1 Hz) coupling constants [42,43]. In the TOCSY spectrum, correlations were visible between H-1 and H-2,H-3,H-4, and the other proton signals were assigned by connectivities identified in the ROESY (strong H-3/H-5) and COSY spectra. The α-configuration of Gal*p*N **A** was proven by the *intra*-residue H-1,H-2 connectivity observed in the ROESY spectrum. In addition, a *N*-acetamido sugar was confirmed by correlation of H-2 at δ 4.35 to the corresponding carbon-bearing nitrogen at δ 49.15, as revealed by the HSQC experiment. The β-anomeric configuration of Gal*p* **D** was inferred from the ^1^*J*_C,H_ coupling constant (162 Hz) [43] and confirmed by the *intra*-residue H-1,H-3 and H-1,H-5 correlations observed in the ROESY spectrum.

The ring *gluco* configuration of the spin system **C** was assigned according to the large *J*_2,3_, *J*_3,4_, and *J*_4,5_ coupling constant values (~10 Hz), and was confirmed by correlations of each H-1, to H-6 with all other protons of the residue in the TOCSY spectrum [39]. Moreover, the α-anomeric configuration of Qui3N **C** was also inferred from the ^1^*J*_C,H_ coupling constants (173–174 Hz), and from the *intra*-residue H-1,H-2 correlation observed in the ROESY spectrum. The site of attachment of the amide-bond acetyl group to Qui3N **C** was confirmed by the correlation of its C-1 with H-3 of the carbon-bearing nitrogen at δ_C_/δ_H_ 175.4/4.33.

The *manno* configuration of **B** was indicated by relatively high coupling constant values of ^3^*J*_3,4_ and ^3^*J*_4,5_ (~10 Hz) contrasting with the small value of ^3^*J*_2,3_ ~3.5 Hz [39,44]. For the *manno* spin system cross-peaks between H-1 and H-2 and H-2 and H-3-H-6, as well as between H-6 and H-1-H-4, were observed in the TOCSY spectrum. The α-configuration of Rha*p* **B** was also proven by the *intra*-residue H-1,H-2 connectivity observed in the ROESY spectrum [41].

Low-field positions of the signals for C-3 of α-Gal*p*NAc **A** (δ 78.33), C-3 of α-Rha*p* **B** (δ 77.14), C-4 of α-Qui*p*3NAc **C** at δ 77.44 and C-4 of β-Gal*p* **D** at δ 77.04, as compared with the chemical shifts of the corresponding non-substituted monosaccharides, elucidated the glycosylation pattern of the sugar residues [37,45].

**Figure 5:**
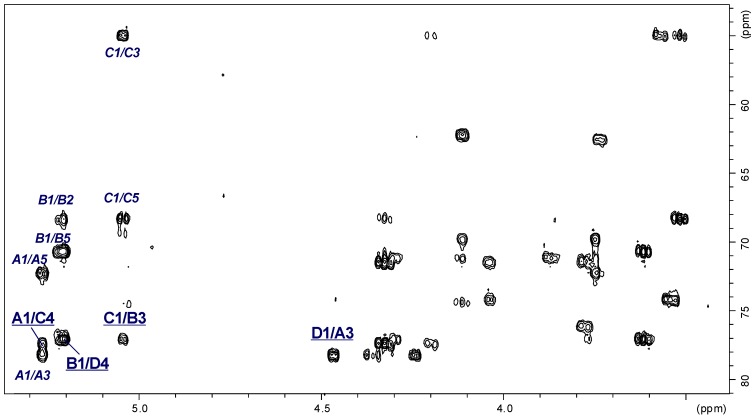
Section of the ^1^H,^13^C HMBC spectrum of the OPS of *A. veronii* strain Bs19. Correlations between anomeric protons and carbons at the glycosidic linkages are underlined. Some other H/C correlations are also depicted (*italic*). Capital letters and Arabic numerals refer to atoms in the sugar residues denoted as shown in Table 3.

The sequence of the sugar residues in the repeating unit was determined by ^1^H,^1^H ROESY and ^1^H,^13^C HMBC experiments. In the 2D ROESY spectrum of the OPS, the following strong NOE contacts were observed: α-Gal*p*NAc H-1 (**A**), α-Qui*p*3NAc H-4 (**C**) at δ 5.27/3.52; α-Qui*p*3NAc H-1 (**C**), α-Rha*p* H-3 (**B**) at δ 5.05/3.87; α-Rha*p* H-1 (**B**), β-Gal*p* H-4 (**D**) at δ 5.21/4.03; and β-Gal*p* H-1 (**D**), α-Gal*p*NAc H-3 (**A**) at δ 4.47/3.95. These data were confirmed by a 2D H-detected heteronuclear multiple-bond (^1^H,^13^C HMBC) experiment (Figure 5), which showed the following *inter*-residue cross-peaks: α-Gal*p*NAc H-1 (**A**), α-Qui*p*3NAc C-4 (**C**) at δ 5.27/77.44; α-Qui*p*3NAc H-1 (**C**), α-Rha*p* C-3 (**B**) at δ 5.05/77.14; α-Rha*p* H-1 (**B**), β-Gal*p* C-4 (**D**) at δ 5.21/77.04; and β-Gal*p* H-1 (**D**), α-Gal*p*NAc C-3 (**A**) at δ 4.47/78.33.

The data presented above were consistent with mass spectrometry analysis of the lower molecular mass fraction of degraded polysaccharide (PS) isolated from the water-soluble LPS after mild acid hydrolysis and separation using GPC.

**Figure 6:**
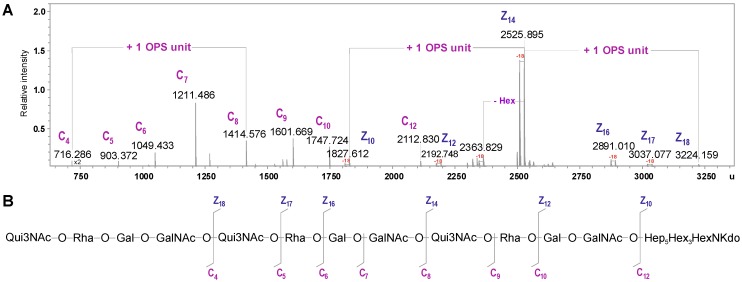
(**A**) Part of the charge-deconvoluted ESI FT-ICR mass spectrum (negative ion mode) of the lower molecular mass fraction of the degraded PS isolated from the LPS of *A. veronii* Bs19, recorded with unspecific fragmentation. (**B**) Fragmentation scheme of the molecule. Mass numbers given refer to the monoisotopic masses. Mass fragments (marked with capital letters) are labeled according to the nomenclature of Domon and Costello [33].

The charge-deconvoluted ESI FT-ICR mass spectrum obtained with unspecific fragmentation (collision voltage 5 V) resulted in the cleavage of glycosidic linkages and yielded Z and C series of mass fragments, which contained the reducing and terminal end of the molecule, respectively. The Z mass fragments, seen as the pairs of mass peaks that differed by Δm = 18 u (loss of water), were attributed to the Kdo containing part of the molecule. As already described, this was expected due to the formation of *anhydro*-Kdo forms during mild acid hydrolysis [46]. In particular, Z_10_, Z_14_, and Z_18_ mass fragments at 1827.612, 2525.895 and 3224.159 u corresponded to the calculated molecular mass of the core oligosaccharide (Hep_5_Hex_3_HexNKdo-H_2_O) without or with one and two OPS repeats attached, respectively. In turn, the C series of fragments were ascribed to the O-repeats containing part of the molecule. In detail, the C_4_ mass fragment at 716.286 u referred to the molecular mass of one OPS repeat (6dHexNAc6dHexHexHexNAc), in turn the C_5_-C_12_ fragments allowed to follow up the sequence of the sugar residues. In summary, the C and Z series of mass peaks confirmed the structure of the OPS established by NMR and enabled to propose the composition of its biological repeating unit. The MS results are shown below (Figure 6, Table 4).

**Table 4:** Composition of the main species present in the charge deconvoluted ESI FT-ICR MS (negative ion mode) of the lower molecular mass fraction of the degraded PS isolated from the LPS of *A. veronii* Bs19, recorded with unspecific fragmentation. Mass fragments (marked with capital letters) are labeled according to the nomenclature of Domon and Costello [33].

Species	M_measured_	M_calculated_	Composition
**C_4_**	716.286	716.284	[6dHexNAc6dHexHexHexNAc]
**C_5_**	903.372	903.369	[6dHexNAc6dHexHexHexNAc]
**C_6_**	1049.433	1049.427	[6dHexNAc6dHexHexHexNAc]6dHexNAc6dHex
**C_7_**	1211.486	1211.479	[6dHexNAc6dHexHexHexNAc]6dHexNAc6dHexHex
**C_8_**	1414.576	1414.558	[6dHexNAc6dHexHexHexNAc]_2_
**C_9_**	1601.669	1601.643	[6dHexNAc6dHexHexHexNAc]_2_6dHexNAc
**C_10_**	1747.724	1747.700	[6dHexNAc6dHexHexHexNAc]_2_6dHexNAc6dHex
**C_12_**	2112.830	2112.831	[6dHexNAc6dHexHexHexNAc]_3_
**Z_10_**	1827.612	1827.603	Hep_5_Hex_3_HexNKdo-H_2_O
**Z_12_**	2192.748	2192.734	[HexHexNAc]Hep_5_Hex_3_HexNKdo-H_2_O
**Z_14_**	2525.895	2525.876	[6dHexNAc6dHexHexHexNAc]Hep_5_Hex_3_HexNKdo-H_2_O
**Z_16_**	2891.010	2891.007	[6dHexNAc6dHexHex2HexNAc2]Hep_5_Hex_3_HexNKdo-H_2_O
**Z_17_**	3037.077	3037.065	[6dHexNAc6dHex2Hex2HexNAc2]Hep_5_Hex_3_HexNKdo-H_2_O
**Z_18_**	3224.159	3224.145	[6dHexNAc6dHexHexHexNAc]_2_Hep_5_Hex_3_HexNKdo-H_2_O

On the basis of all the data obtained, it was concluded that the biological repeating unit of the OPS from *A. veronii* strain Bs19, serotype O16 had the structure:

→4)-α-d-Qui*p*3NAc-(1→3)-α-l-Rha*p*-(1→4)-β-d-Gal*p*-(1→3)-α-d-Gal*p*NAc-(1→

The structure of the OPS from *A. veronii* Bs19 described herein is the first established for the species. A similar tetrasaccharidic backbone, in respect to both sugar sequence and glycosylation pattern, has been identified as the main component of the O-antigen repeating units of *Salmonella* Dakar, serogroup O28, and *Escherichia coli*, serogroup O71 [47,48]. Whereas, in *E. coli* O71, the difference is caused by non-stoichiometric *O*-acetylation of β-d-Gal*p*, in the OPS of *S.* Dakar, α-d-Gal*p*NAc is additionally substituted at O-4 by β-d-Glc*p* [47,48]. It is likely that this terminal residue is also responsible for structural differences within the serogroup O28. Some aspects of the molecular basis for subdividing the O28 serogroup of the Kauffmann-White scheme into three subfactors O28, O28_1_ and O28_2_ has been recently explained [49]. Studies with monoclonal antibodies confirmed that the O28_1_—antigen specificity is attributed to the 3-substituted or 3,4-disubstituted α-d-Gal*p*NAc, which constitute the component of the main chain both *S.* Dakar and *Salmonella* Telaviv OPSs [49].

## 3. Experimental Section

### 3.1. Bacterial Strain, Cultivation Conditions and Isolation of the LPS

The *A. veronii* strain Bs19 was isolated from pathologically altered skin of carp suffering from ulcerative syndrome in a commercial pond, as previously reported [31], and was obtained from the Collection of the Microorganisms of the Department of Fish Diseases, National Veterinary Research Institute (Pulawy, Poland). Based on both biochemical properties and PCR-RFLP analysis of the 16S rDNA, strain Bs19 was identified to the species level, and classified to the serogroup O16 [28] according to the scheme of Sakazaki and Shimada [18].

The bacteria were cultivated in tryptic soy broth (TSB) at 28 °C for 72 h. The cells were harvested by low speed centrifugation (8500× *g*, 20 min). The recovered bacterial cell pellet was washed twice with 0.5 M saline and once more with distilled water. Bacterial cells were digested with lysozyme, RNAse, and DNAse (24 h, 1 mg/g), and then with Proteinase K (36 h, 1 mg/g) in 50 mM phosphate buffer (pH 7.0) containing 5 mM MgCl_2_. The suspension was dialyzed against distilled water and freeze-dried. The digested cells were extracted three times with aq 45% phenol at 68 °C [32], and the separated layers were dialyzed against deionized water, purified by ultracentrifugation (105,000× *g*, 4 h) and freeze-dried to give LPS in a yield of 4.5% of dry bacterial cell mass. In total, 890 mg of LPS were isolated, 93.3% of which was *S*-form (water phase) and 6.7% *SR*- and *R*-form (phenol phase). Both LPS preparations were further analyzed. 

### 3.2. Isolation of the OPS

The OPS was obtained by mild acid hydrolysis of the *S*-form LPS (100 mg) with 2% acetic acid at 100 °C for 3 h, followed by GPC of the water soluble-portion on a column (1.8 × 80 cm) of Sephadex G-50 fine (Pharmacia, Sweden) using 1% acetic acid as an eluent and monitoring with a Knauer differential refractometer (Knauer, Berlin, Germany). The yield of the OPS fraction was 24.5% of the LPS mass subjected to hydrolysis. The sediment released by acid hydrolysis of the LPS was purified by the Bligh-Dyer method as it was described earlier [50] to give 12 mg lipid A.

### 3.3. Chemical Analyses

For neutral and amino sugar analysis, the LPS samples and the OPS were hydrolyzed with 2 M CF_3_CO_2_H (120 °C, 2 h), *N*-acetylated, reduced with NaBD_4_ and acetylated with a 1:1 pyridine-acetic anhydride mixture (100 °C, 30 min). To release acidic sugar components, LPSs and the OPS were subjected to methanolysis (1 M HCl in methanol, 85 °C, 16 h), carboxyl reduction with NaBD_4_ in aqueous 50% methanol, hydrolysis with 2 M CF_3_CO_2_H and acetylation. The products were identified as alditol acetates by GC-MS [51] on a Hewlett-Packard HP5890A-HP5971 instrument equipped with an HP-5ms (SLB-5ms) capillary column (30 m × 0.25 mm; Supelco, St. Louis, MO, USA), applying a temperature gradient of 150 °C (5 min) to 310 °C at 5 °C min^−1^.

The absolute configuration of monosaccharides was determined by GC of acetylated (*S*)-2-butyl glycosides using authentic sugars as standards [36].

Methylation of the OPS was performed by the procedure of Hakomori [52]. The permethylated OPS was subjected to hydrolysis in 2 M CF_3_CO_2_H (120 °C, 2 h), *N*-acetylation, and reduction with NaBD_4_. Partially methylated alditols (PMAA) were converted into acetate derivatives, and analyzed by GC-MS as above.

For fatty acid analysis, a sample of the lipid A (1 mg) was subjected to methanolysis in 2 M methanolic HCl (85 °C, 12 h). The resulting fatty acid methyl esters were extracted with hexane and converted to their *O*-trimethylsilyl (*O*-TMS) derivatives, as described [50,53]. The methanol layer, containing the methyl glycosides was dried and acetylated with pyridine-acetic anhydride mixture. The fatty acid derivatives, as well as acetylated methyl glycosides were analyzed by GC-MS as above.

### 3.4. NMR Spectroscopy

1D ^1^H NMR, ^13^C NMR and 2D NMR experiments were recorded in a D_2_O solution at 32 °C using a Bruker Avance III 700 MHz spectrometer (operating frequencies 700.43 MHz for ^1^H NMR and 176.14 MHz for ^13^C NMR) and applying standard Bruker software (Bruker, TopSpi, Rheinstetten, Germany). Chemical shifts were reported relative to external acetone as reference (δ_H_ 2.225, δ_C_ 31.45). The following homo- and heteronuclear correlated two-dimensional spectra were used for general assignments: ^1^H,^1^H DQF-COSY, TOCSY, ROESY, ^1^H,^13^C HSQC, and ^1^H,^13^C HMBC.

### 3.5. Mass Spectrometry Analysis

ESI FT-ICR MS was performed in negative ion mode using a hybrid Apex Qe FT-ICR MS instrument (Bruker Daltonics), equipped with a 7 Tesla actively shielded magnet and an Apollo dual ion source. Samples (~10 ng μL^−1^) were sprayed at a flow rate of 2 μL min^−1^. Capillary entrance voltage was set to 3.8 kV, and dry gas temperature to 200 °C. For unspecific fragmentation the DC offset (collision voltage) of the collision cell was set from 5 V to 30 V. Under these conditions the labile linkage between the lipid A and the core oligosaccharide is cleaved [54,55] resulting in intensive Y^−^ and B^−^ fragments representing the lipid A and the core oligosaccharide moieties (according to the nomenclature of Domon and Costello [33]). The mass spectra were charge deconvoluted and mass values given in all spectra and tables refer to the monoisotopic signal of the neutral molecules. Mass calibration was done externally by well-characterized LPS of known structure.

### 3.6. SDS-PAGE

LPS preparations were separated in 12.5% SDS-Tricine polyacrylamide electrophoresis gel and bands were visualized by silver staining after oxidation with periodate [56].

## 4. Conclusions

*Aeromonas* sp. bacteria are common inhabitants of aquatic environments and have been described in relation to fish and human diseases, particularly food-borne associated [2,18]. The cell envelope of *Aeromonas*, as that of other Gram-negative bacteria, contains LPS, a crucial amphiphilic and immunodominant constituent of the outer membrane [57]. The OPS of the LPS is one of the most variable components on the cell surface, providing the basis for serotyping of bacteria. As it was shown that specific *Aeromonas*, as well as *Cronobacter* O serotypes, were associated with enteritis epidemics [5,58], especially in children, it is important to identify epidemiologically relevant strains and to understand the immunochemical aspects of antigen specificity within the serogroups.

While most OPS are distinct among different genera, the core region of LPS, in particular the inner part, tends to be conserved within a genus or even family. The fact that the inner core of LPS from distantly related bacteria shares structural features is a reflection of evolutionary relationship and the importance of this region in outer membrane integrity [57]. On the other hand, the outer core shows more structural diversity, as might be expected for a region exposed to the selective pressures of host responses, location of bacteriophage receptors, and environmental stress. Recently, the complete core structure of LPS from *A. hydrophila* strain AH-901, which is a mutant in a gene encoding mannose transferase, has been published. The core nonasaccharide was composed of two d,d-Hep residues in combination with four l,d-Hep. Two other sugars, *i.e.*, β-glucose and α-glucosamine, as well as one residue of α-3-deoxy-d-*manno*-oct-2-ulosonic acid, at the reducing end, were also detected. Additionally, one of d,d-heptose residue was non-stoichiometrically substituted with β-galactopyranose. No charged groups were reported except for one phosphate group at the 4-position of the Kdo unit [59,60].

Moreover, our latest findings revealed that the core oligosaccharide with the prevalence of heptose residues and the composition Hep_6_Hex_1_HexN_1_Kdo_1_P_1_ represents a structure shared by LPS core part of the strains belonging to the species *A. hydrophila* and *A. bestiarum* [27,59,60].

Interestingly, our present results show that the core region may vary to some extent within *Aeromonas* spp. bacteria. In this work, water- and phenol-soluble LPS preparations isolated from *A. veronii* strain Bs19 have been structurally characterized. Compositional analysis identified d,d-Hep and l,d-Hep, and ESI MS experiments confirmed that the core decasaccharide had a different structure than those established earlier [27,59,60], namely Hep_5_Hex_3_HexN_1_Kdo_1_P_1_. Some differences were also noticed in the composition and acylation pattern of the lipid A. Although LPS glycoforms had tetra-acylated and hexa-acylated lipid A species with amide-linked 14:0(3-OH) and a backbone comprising a bisphosphorylated GlcN disaccharide, an AraN residue was exclusively detected as a non-stoichiometric substituent in the lipid A from LPS of the phenol-soluble fraction. Additionally, some lipid A species contained ester-linked 12:0 and 14:0.

In the future, we would like to focus on structural studies of LPS heterogeneity within the strains belonging to the group *Aeromonas sobria* complex and combine the data with the elucidation of the location and organization of LPS gene clusters.

## Abbreviations

NMR: nuclear magnetic resonance
ESI-MS: electrospray ionization mass spectrometry
FT-ICR: Fourier transform ion cyclotron resonance
OPS: *O*-specific polysaccharide
ADP: adenosine diphosphate
SDS-PAGE: sodium dodecyl sulfate polyacrylamide gel electrophoresis
ESI-FT-ICR: electrospray ionization Fourier transform ion cyclotron resonance
GC-MS: gas chromatography with mass spectrometry
DQF-COSY: double quantum filtered correlation spectroscopy
NOE: Nuclear Overhauser effect
ROESY: rotating frame Overhauser effect spectroscopy
TOCSY: total correlation spectroscopy
PCR-RFLP: polymerase chain reaction/restriction fragment length polymorphism
