# Correction: Structural Studies of the Lipopolysaccharide from the Fish Pathogen *Aeromonas veronii* Strain Bs19, Serotype O16. *Mar. Drugs* 2014, *12*, 1298–1316

**DOI:** 10.3390/md12094741

**Published:** 2014-09-05

**Authors:** Anna Turska-Szewczuk, Katarzyna A. Duda, Dominik Schwudke, Agnieszka Pekala, Alicja Kozinska, Otto Holst

**Affiliations:** 1 Department of Genetics and Microbiology, M. Curie-Sklodowska University, Akademicka 19, Lublin 20-033, Poland; 2 Division of Structural Biochemistry, Research Center Borstel, Leibniz-Center for Medicine and Biosciences, Parkallee 4a/c, Borstel D-23845, Germany; E-Mails: kduda@fz-borstel.de (K.A.D.); oholst@fz-borstel.de (O.H.); 3 Division of Bioanalytical Chemistry, Research Center Borstel, Leibniz-Center for Medicine and Biosciences, Parkallee 10, Borstel D-23845, Germany; E-Mail: dschwudke@fz-borstel.de; 4 Department of Fish Diseases, National Veterinary Research Institute, Partyzantow 57, Pulawy 24-100, Poland; E-Mails: a.pekala@piwet.pulawy.pl (A.P.); koala@piwet.pulawy.pl (A.K.)

We found one editorial mistake in our published paper [1]. In Line 2 of Table 4, the same composition of sugars is given for the C4 and C5 species (in the C5 species, one residue: 6dHexNAc has been missed). The correct composition for the C5 species is: [6dHexNAc6dHexHexHexNAc]6dHexNAc. We apologize for any inconvenience caused to our readers. The corrected table is as follows:

**Table 4:** Composition of the main species present in the charge deconvoluted ESI FT-ICR MS (negative ion mode) of the lower molecular mass fraction of the degraded PS isolated from the LPS of *A. veronii* Bs19, recorded with unspecific fragmentation. Mass fragments (marked with capital letters) are labeled according to the nomenclature of Domon and Costello [33].

Species	M_measured_	M_calculated_	Composition
**C_4_**	716.286	716.284	[6dHexNAc6dHexHexHexNAc]
**C_5_**	903.372	903.369	[6dHexNAc6dHexHexHexNAc]6dHexNAc
**C_6_**	1049.433	1049.427	[6dHexNAc6dHexHexHexNAc]6dHexNAc6dHex
**C_7_**	1211.486	1211.479	[6dHexNAc6dHexHexHexNAc]6dHexNAc6dHexHex
**C_8_**	1414.576	1414.558	[6dHexNAc6dHexHexHexNAc]_2_
**C_9_**	1601.669	1601.643	[6dHexNAc6dHexHexHexNAc]_2_6dHexNAc
**C_10_**	1747.724	1747.700	[6dHexNAc6dHexHexHexNAc]_2_6dHexNAc6dHex
**C_12_**	2112.830	2112.831	[6dHexNAc6dHexHexHexNAc]_3_
**Z_10_**	1827.612	1827.603	Hep_5_Hex_3_HexNKdo-H_2_O
**Z_12_**	2192.748	2192.734	[HexHexNAc]Hep_5_Hex_3_HexNKdo-H_2_O
**Z_14_**	2525.895	2525.876	[6dHexNAc6dHexHexHexNAc]Hep_5_Hex_3_HexNKdo-H_2_O
**Z_16_**	2891.010	2891.007	[6dHexNAc6dHexHex_2_HexNAc_2_]Hep_5_Hex_3_HexNKdo-H_2_O
**Z_17_**	3037.077	3037.065	[6dHexNAc6dHex_2_Hex_2_HexNAc_2_]Hep_5_Hex_3_HexNKdo-H_2_O
**Z_18_**	3224.159	3224.145	[6dHexNAc6dHexHexHexNAc]_2_Hep_5_Hex_3_HexNKdo-H_2_O

## References

[1] Turska-Szewczuk A., Duda K.A., Schwudke D., Pekala A., Kozinska A., Holst O. (2014). Structural Studies of the Lipopolysaccharide from the Fish Pathogen *Aeromonas veronii* Strain Bs19, Serotype O16. Mar. Drugs.

